# Identifying Spatial Patterns and Ecosystem Service Delivery of Nature-Based Solutions

**DOI:** 10.1007/s00267-022-01613-y

**Published:** 2022-03-09

**Authors:** Paulina Guerrero, Dagmar Haase, Christian Albert

**Affiliations:** 1 Institute of Geography, Chair for Environmental Analysis and Planning in Metropolitan Regions, Ruhr University Bochum, Universitaetsstr. 150, 44780 Bochum, Germany; 2 Department of Geography, Humboldt University of Berlin, Rudower Chausee 16, 10099 Berlin, Germany; 3 Department of Computational Landscape Ecology, Helmholtz Centre for Environmental Research—UFZ, Permoser Str. 15, 04318 Leipzig, Germany

**Keywords:** Ecosystem services, Rivers, Spatial analysis, NBS, GIS

## Abstract

Compared to technical infrastructure, nature-based solutions, NBS, strive to work with nature and to move beyond business-as-usual practices in order to address societal challenges such as flood risks. This research aims to spatially identify possible NBS areas and evaluate the areas capacity to provide selected ecosystem services, ES, for the Lahn river landscape in Germany. The research follows the functional landscape approach using hydromorphological landscape units, HLU, based on specific biophysical spatial criteria, such as slope, to then identify locations which may be considered suitable for NBS. The current ES delivery of these possible NBS areas is then evaluated. The three ES assessed are carbon storage, nutrient retention and recreation. We then undertake a geospatial comparison analysis to show the spatial relationships and patterns that emerge in regards to the ES configuration of the distinct NBS apt areas. Results show the HLU method serves to delineate and identify areas where NBS may exist or be implemented. The data depicts a distinct spatial pattern for each possible NBS space and complementary ES delivery. This explorative method is a useful spatial approach that can support NBS implementation and serve to investigate the multiple benefits NBS provide. The use of ecosystem services to compare and understand NBS is a viable prospect that must, however, be cautiously, locally and scientifically approached. Noticeable limitations regarding ES assessment remain, as available methods are often insufficiently inclusive of natural ecosystem processes and functions. Further research should assess a broader spectrum of NBS and their delivery of ES.

## Introduction

Rivers have been referred to as our planet’s bloodlines. We depend on them for the well-being of our ecosystems and our societies. Rivers are the sources of innumerable ecosystem services, such as clean drinking water, climate regulation and outlets for recreational opportunities (Aylward et al. 2005; Hanna et al. 2018). Unfortunately, rivers and their floodplains throughout the world are continually facing intense and multiple pressures including contamination, over-consumption of water and biodiversity loss which is only expected to escalate in the coming years (Palmer and Bernhardt 2006; Schindler et al. 2016; WWAP 2018). We need to urgently find solutions and ways to manage competing demands while protecting our valuable freshwater resources.

For decades, technical measures, such as large dams, canalization and levees, were seen as archetypical measures for rivers the world over (van Vuren et al. 2014; WCD 2000; Woo 2010). We now know that these structures in reality often exacerbate the problems they were meant to solve, as they often create undesirable side effects and result in negative consequences for the local ecosystem and communities (Keesstra et al. 2018; Roni et al. 2008; Van Wesenbeeck et al. 2014; Vörösmarty et al. 2010; Woo 2010; WWAP 2018). In light of this background, nature-based solutions (NBS) have been presented as a way forward.

Nature-based solutions, including the extension of floodplains and wetland creation and protection, have gained considerable momentum and attention in the past years (Albert et al. 2019; Dushkova and Haase 2020; Thorslund et al. 2017). Even though the concept has long been studied in theory and practice in fields such as environmental engineering and river rehabilitation, this new impulse considers a more inclusive, transdisciplinary approach to the topic and aims to include more views and voices in order to move this notion forward (Cohen-Shacham et al. 2016; Eggermont et al. 2015; Roni et al. 2008). The European Commission (EC) presents NBS as solutions that “harness the power and sophistication of nature to turn environmental, social and economic challenges into innovation opportunities” (EEA 2015). The International Union for Conservation of Nature, IUCN, another leading voice, adds that NBS are actions “to protect, sustainably manage and restore natural or modified ecosystems” and that these solutions “deliver clear biodiversity benefits in terms of diverse, well-managed and functioning ecosystems” (Cohen-Shacham et al. 2016). While the descriptor NBS is considered an umbrella term that allows for acceptance and advancement of the concept (Cohen-Shacham et al. 2016; Nature 2017; Schanze 2017), for water NBS a more sharpened definition comes from UN Water stating that water NBS are characterized by “whether natural processes are being proactively managed” and that ecosystem services are used, or harnessed, in order to achieve water-related objectives (WWAP 2018).

In line with the previous definition, ecosystem services have been heralded as useful instruments to inform NBS (Albert et al. 2019; Keesstra et al. 2018; Nesshöver et al. 2017). ES may be seen as a potential “common currency” for the evaluation of NBS and drawing upon ES assessments to evaluate the “consequences of differing solutions” may provide a deeper, more concrete understanding of how NBS fare (Nesshöver et al. 2017). The notion that NBS should be multi-functioning and providers of multiple benefits also aligns with the core of the ES approach to disclose these benefits (Nesshöver et al. 2017; Pontee et al. 2016; Raymond et al. 2017; Thorslund et al. 2017). Ecosystem services can provide a numerical value of a provision, for example carbon sequestration, and thus, may be potentially valuable to allow for a comparison of distinct solutions. However, there remains a gap between this potential connection and the actual knowledge and sound research of how ES may be used to analyze the impact and effectiveness of NBS (Kabisch et al. 2016).

Research in the field of ecosystem service assessment and landscape planning has looked to find and define working units that allow for a deeper understanding of the relationship between ES and spatiotemporal factors, structures and dynamics (Andersson et al. 2015; Syrbe and Walz 2012). Some of these working units include service providing units (SPUs) which stemmed from landscape ecology to represent a “collection of individuals from a given species and their characteristics necessary to deliver an ecosystem service”; while Andersson later defined SPUs on a broader scope to be the “smallest distinct physical unit that generates a particular ES and is addressable by planning and management” (Andersson et al. 2015; Luck et al. 2009). Service providing areas (SPAs), another unit for ES assessment, strongly rely on spatial components and serve as the areal basis for service provision (Fisher et al. 2009; Syrbe and Walz 2012). While these approaches of how to pin-point ES provision have advanced the field of ES assessment significantly, there are still vast challenges that have not been resolved and many methods have weak links to ecosystem processes and a lack of biophysical realism (Lavorel et al. 2017; Seppelt et al. 2011). Considering rivers, ES assessments often fail to take into account the natural, biophysical compositions of rivers, including geomorphic, hydrologic, and biological parameters (Tomscha et al. 2017).

Building upon the notion that ES may serve as instruments to promote and operationalize NBS, this paper is inspired by hydrological modeling standards of delineating specific units based solely on biophysical characteristics and looks to incorporate these concepts into NBS. In line with the call to consider more biophysical determinants and “fundamental river-floodplain” behavior in ES assessments and instead of working with human-defined or mixed units, such as SPAs and SPUs, we see an opportunity to further advance the concept by creating units based solely on natural determinants for river NBS identification and then using these units in combination with ES assessment (Haase and Gläser 2009; Lavorel et al. 2017; Tomscha et al. 2017). These units are referred to as hydromorphological land units, or HLU, which are based on biophysical parameters relating to NBS location types, such as wetlands and floodplains (Guerrero et al. 2018). Considering research on NBS and ES has recently advanced dynamically, a knowledge gap still exists regarding how to properly utilize ecosystem service assessments to support the planning and implementation of NBS.

To take on this research gap, we explore how to combine the knowledge and methods in the fields of hydrological and ES modeling order to support action and implementation of NBS. We aim to: identify areas where specific NBS either already exist or could be developed based on natural characteristics, to assess those areas with regard to their delivery of selected ecosystem services and to explore geospatial and statistical patterns of these units in relation to each other. We look to answer the following questions: (1) what is the current state of ES delivery of the identified areas?, and (2) how do these identified units relate to one another in regards to their actual ES delivery, both spatially and quantitatively?

## Case Study

**Fig. 1:**
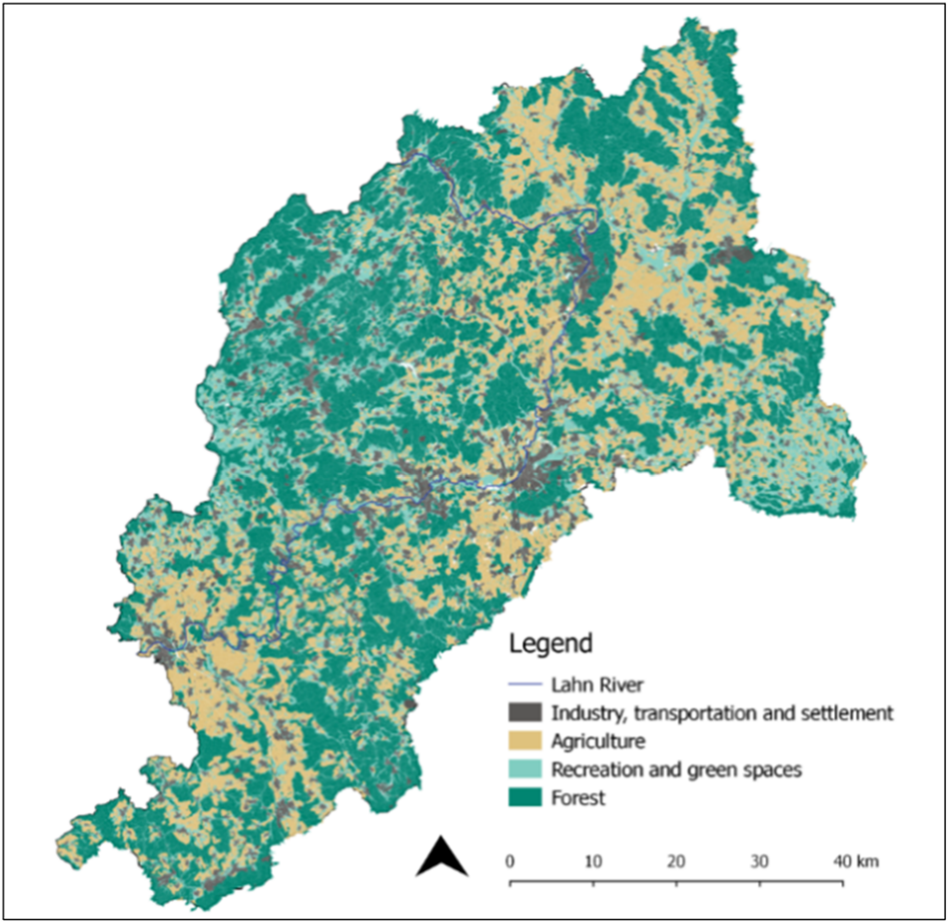
Map of general land uses in the Lahn River in Hesse, adapted from Guerrero et al. (2018)

The focus of this study is the Lahn River landscape in Hesse, Germany. With a total catchment area of 5931 km^2^, the Lahn River is a vital tributary of the river Rhine and runs through three states in Germany. This study centers on the river stretch in the federal state of Hesse, as the majority of its course is found in the state of Hesse (4757 km^2^). Our analysis concentrated on a 5 km perimeter around the river in order to be inclusive of bankfull depth criteria and floodplain valley morphology (Ward et al. 2016). According to Martin (2004) land use of the Lahn watershed consists of 70% agricultural, 16% forest, and 14% urban. The floodplain of the Lahn is primarily cultivated or used for grazing and reaches a maximum width of 2.5 km downriver from Giessen; hillslopes in the study reach are generally forested (Martin 2004). See Fig. 1 for a general land uses map of the study region (Fig. 1). For a location map of the river, we refer the reader to the insert map in Fig. 3.

The Lahn River, according to the profiles of German stream types, is a type 9.2 large highland river exhibiting a very dynamic flow with high discharge fluctuations, high habitat diversity, and pronounced discharge events (Pottgiesser and Sommerhäuser 2004). A large part of the Lahn’s floodplains has a status ranging from “significantly modified” up to “heavily modified” (EU LIFE 2014). This is indicative of a river whose course, flow, and natural dynamics have been significantly affected. The numerous dams and locks located in the lower part of the Lahn River allow for shipping transport; yet, simultaneously, these structures hinder fish migrations, such as that of the salmon, and create complications for kayakers and other recreational users (Von Keitz 2015).

Additionally, the Lahn River is considered a spearheading project aiming to develop integrated concepts for the future development of rivers, as suggested by the Blue Belt Program of the German government (BMVI 2017; EU LIFE 2014). The Living Lahn Program, a recipient of a 10-year EU Life grant, has the specific goal to improve the ecological state of the river and ensure it is liveable for all stakeholders (Von Keitz 2015). The parallels of this river to others rivers in Germany in a similar situations, makes this an adequate case for a possible transferability of methods. For these reasons, this case study provides a window for NBS implementation and serves as a fitting test case for our study.

According to the EU’s Water Framework Directive an additional problem that the Lahn faces is its current water quality, currently categorized as a Grade II, or a moderately burdened river, with an ecological status of “unsatisfactory” or “bad” (EU LIFE 2014). A reason for this low water quality is partially traced back to former mining pressures and agricultural loads, with diffuse contaminants, comprising of heavy metals, which filtrate towards the watershed, floodplain and, eventually, the flow of the river itself (EU LIFE 2014; Hahn et al. 2016).

## Methods

In order to assess our case study and have adequate and comparable ES data, first appropriate methods for each specific ecosystem service were undertaken, a spatial delineation of the landscape was performed and the results were consequently statistically and spatially evaluated. All of the spatial analysis was processed using the geographical information system QGIS (QGIS Development Team 2018). The spatial and numerical data used, values and sources can be found in Appendices A–D.

### Ecosystem Services Indicators and Values

Literature-based indicators relevant for rivers and their related methods and data were selected to represent the ecosystem services for this study. Above- and below-carbon storage, nitrogen retention, and outdoor recreation comprise the indicators used to analyze the following riverine ES: carbon storage, nutrient retention, and recreation, correspondingly, see Table 1. Our priority for ES selection was to use established and ascertainable ES methods as our research focused on testing the HLU method with sound and balanced ES assessment. Therefore, the selected ES are some of the most frequently assessed riverine ES, include regulating and cultural ES categories allowing for comparison, and are emphasized as being beneficial for well-being (Aylward et al. 2005; Hanna et al. 2018). All of the indicators have been well-tested in other studies and are valued using empirical methods (Fagerholm et al. 2016; Larondelle et al. 2014; Rall et al. 2017; Sharp et al. 2016; Strohbach and Haase 2012; Yan et al. 2018).

**Table 1:** Indicators and methods for the ecosystem services quantified

Ecosystem Service	Indicator	Method
Carbon storage	Above-ground and soil carbon storage	Above-ground carbon storage linked to land use [MgC/ha] according to Strohbach and Haase (2012).
Nutrient retention	Nitrogen retention	InVEST NDR Model (Sharp et al. 2016).
Recreation	Outdoor recreation	Heatmap from Maptionnaire survey results (Fagerholm et al. 2016; Rall et al. 2017).

#### Above-ground and soil carbon storage

The IPCC identifies five types of carbon pools; these are: 1) above-ground biomass, 2) below-ground biomass, 3) dead organic matter: dead wood, 4) dead organic matter: litter, and 5) soil organic matter (SOC) (Paustian et al. 2006). This study focused on the two most relevant pools, above-ground biomass, and soil carbon storage, for the ES assessment of the study area (EPA 2018; Paustian et al. 2006). The values used to quantify the aboveground carbon storage were obtained from an empirical study by Strohbach and Haase (2012). The aforementioned study was carried out in the city of Leipzig, Germany, which due to its similar latitudinal location and vegetation composition as our case study, serves as an ideal data source. Those values which were not available from Strohbach and Haase (2012), such as for agriculture and grassland, were obtained from the German United Nations Framework Convention on Climate Change (UNFCCC) Report (Umweltbundesamt 2018). The soil carbon storage values for urban-related land-uses were adapted from a study from Majidzadeh et al. (2017) which focused on the soil carbon storage of impervious surfaces. Unsealed soil carbon values were derived from German UNFCCC Reports and those of the state of Hesse (HLUG 2005; Umweltbundesamt 2018). All values used, both for above-ground and soil carbon, were differentiated by land-use and used the same metric, see Appendix B.

#### Nitrogen retention

Nutrient loading is the “most prevalent water quality challenge” globally, and it represents increased threats to human health and the environment itself (Van Wesenbeeck et al. 2014). One of nature´s natural purification processes is nutrient retention through vegetation. This occurs when vegetation, especially riparian vegetation, present in the watershed absorbs or transforms some pollutants and acts as a filter before pollutants enter a stream (Thorslund et al. 2017; Yan et al. 2018). Spatially identifying where these areas of natural retention are present is of great interest and remains a challenge for planning sustainable futures (Sharp et al. 2016).

The nitrogen retention ES assessment was performed using the InVEST Nutrient Delivery Ratio (NDR) module developed and made freely available by the Natural Capital Project (www.naturalcapitalproject.org). The model is based on a mass balance approach, focused on the movement of nutrient loads throughout space and represents the long-term, steady-state flow of nutrients through empirical relationships (Idem). Using spatial and quantitative data, each pixel’s load and NDR, which is a function of the upslope area and downslope flow path, are calculated and modeled (Idem). The NDR model returns a pixel-level map of how much load from each pixel can reach the stream (measured in kg N/pixel). The amount of nitrogen that reaches a river is dependent on spatial factors, including flow direction and precipitation which are included as variables in the model. The NDR considers key factors such as topography and biophysical characteristics, and these calculations are done at a watershed level (Idem). For the variables required by the model, we took into account the results and recommendations of the UK review of the NDR InVEST model (Redhead et al. 2018). This paper focused solely on the nitrogen surface flow option of the model, see Appendices B and C for the data sources and values used.

#### Outdoor recreation

To examine the cultural ES recreation, we used public participation GIS (PPGIS) data from a local survey conducted with residents of the Lahn river landscape (Gottwald and Stedman 2020; Verbrugge et al. 2019). PPGIS is a participatory mapping approach usually initiated or conducted by scientists or governments with a specific purpose and emphasis on land use planning and management and has been extensively used for spatial ES mapping and modeling (Brown and Fagerholm 2015; Brown and Kyttä 2014; Kahila and Kyttä 2009). The data was collected through the softGIS online survey tool Maptionnaire (maptionnaire.com), which combines a mapping interface with traditional survey questions and results in geo-referenced data. Thus, the data is spatial in nature and direct from the local residents. The sample group, i.e., the participants, were primarily younger, higher earning, slightly less educated males than the average local population. Hence, the limited representativeness of the entire population needs to be taken into consideration when interpreting the findings (Gottwald and Stedman 2020). Further information and details of the Lahn Maptionnaire survey such as questionnaire design, participant structure, and methodology can be found in Gottwald and Stedman (2020).

This paper focuses on the section of the PPGIS survey where participants were asked to locate points where they use the Lahn river landscape, i.e., activity points (*n* = 310), for further information please see Appendix A in Gottwald and Stedman (2020). For this paper, we use the indicator outdoor recreation for recreation ES and the definition established in PPGIS literature which entails that outdoor recreation consists of activities such as practicing outdoor sports, walking, hiking, biking, dog walking (Aylward et al. 2005; Fagerholm et al. 2016; Plieninger et al. 2013). Therefore, activities where the participant indicated social type of activities, such as meeting friends or barbequing, were not included. Participants could locate activities on the map, which were categorized in the survey as nature, recreation and sport activities, with one draw button for each category. After locating one of these activities, respondents had the opportunity to further specify the exact kind of activity by choosing one or more options from a list of four, five, and eight types of nature, recreation, or sports activities respectively, or adding their own individual activity type, see Appendix D.

The spatial values were mapped using kernel density analysis for the activity points. This is a technique that calculates the density of points around each output raster cell and it is based on the quadratic kernel function (Silverman 1986). It is a method widely used to visualize the spatial patterns of PPGIS based ecosystem service indicators (Brown and Fagerholm 2015). At each point a weighted density from the centroid to a self-defined radius is analyzed and visualized by a smoothed surface around each point; adding the values of all kernel surfaces overlaying each raster cell calculates the density of each raster cell. The radius selected was 5 km which considered: (a) Beichler (2015) who found that recreation had a critical distance, i.e., the distances people are willing to go to experience a cultural ES, of 4 km and, (b) that our area of study of the river is based on a 5 km buffer radius (Beichler 2015).

### Developing Hydromorphological Landscape Units

As previously mentioned, this research relied on hydromorphological land units, HLU, for the spatial identification of NBS types, including wetlands, floodplains, and forest (Guerrero et al. 2018). This method is inspired by hydrological models, such as the Soil and Water Assessment Tool and the Precipitation Runoff Modelling System which work with hydrological response units, HRU (Markstrom et al. 2015; University of Texas AandM 2012). HRU are defined as homogenous units containing the same soil, land use, and slope characteristics, and form the basis for modeling the water balance of watersheds (Kalcic et al. 2015; Schmalz et al. 2015; University of Texas A&M 2012). Thus, the HRU delineation method identifies areas of similar hydrologic behavior for each land unit. In comparison to a HRU, which has a hydrological focus and incorporates land use as a criterion, a hydromorphological land unit consists of the critical biophysical parameters specific to characterize a natural feature. This identification of homogenous spatial units with similar behaviors was the inspiration behind Guerrero et al. (2018) for the NBS type relating to floodplains; this method is expanded to include wetland and forest HLU.

**Fig. 2:**
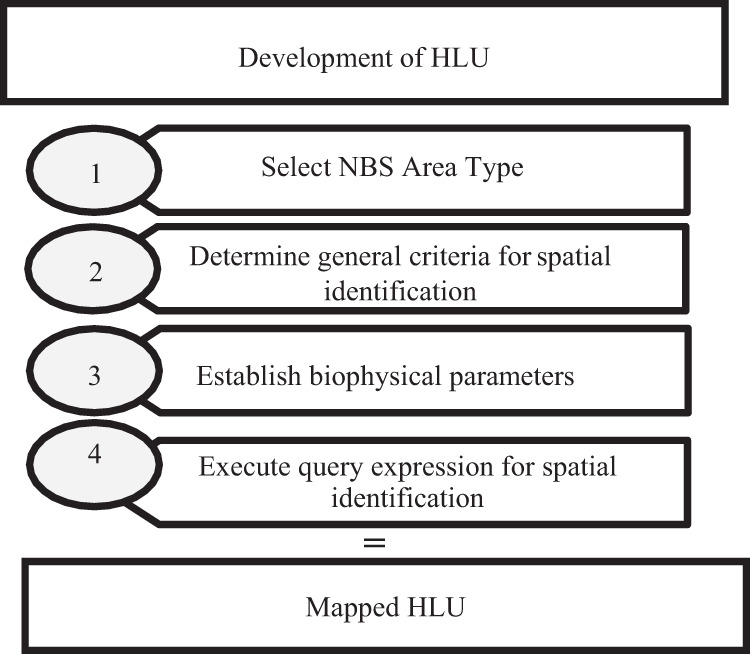
Methodological process for HLU spatial development (adapted from Guerrero et al. 2018)

These criteria and parameters for wetlands, floodplains, and forest, are defined based on established literature and with consideration that the parameters be spatially identifiable. Once the criteria and parameters for the HLU were determined for each natural feature, we then ran spatial queries using these parameters, i.e., GIS calculations, for the corresponding thematic spatial layers. The resulting layers were then processed, intersected, and joined, see Fig. 2. The spatial combination of these criteria and parameters creates uniform units specific to each natural terrain.

These HLU units can be considered as “opportunity spaces for NBS safeguarding and/or development.” As such, the opportunity spaces may include both areas with opportunities for safeguarding already existing, working NBS areas as well as areas where new NBS can be established.

Table 2 details each of the criteria and parameters used for the HLU delineation. Each parameter was carefully selected based on well-established literature specific to each type of natural landscape. While standard or established criteria do not exist, there is ample research and literature that has specified minimum and/or critical requirements (NAS 2002; NRC 1995). For example, for wetland identification, the presence of hydric soils and hydrophytic vegetation, is paramount to the presence of a wetland area (NRC 1995; Tiner 2012). The wetland HLU were excluded from the floodplain HLU, in order to not double count these areas. In addition to the specific soil types and slope parameters, the NBS type Forest was approached with the assumptions that: (1) the established wetland and floodplain areas could not be forests, and (2) since we aimed to include only biophysical rather than human constraints, the remaining area was assumed to have once held forest growth (Haase 2003; Haase and Gläser 2009).

**Table 2:** Criteria and biophysical parameters for wetland, floodplain and forest HLU

HLU	Data Layer	Parameters	Sources for parameters
Wetland	DEM	Slope ≤ 0.02	(NRC 1995)
	Soil Map	Hydric soils	(NAS 2002; NRC 1995)
	Vegetation	Hydrophytic vegetation	(Bundesamt für Naturschutz 2020; NAS 2002; NRC 1995; Tiner 2012)
	Groundwater Measurements	Water Table Depth ≤ 2.0 m	(NRC 1995)
Floodplain	DEM	Slope ≤ 0.03	(Haase 2003; Haase and Gläser 2009; Tockner et al. 2005; Ward et al. 2016)
	Soil Map	Organic and Alluvial	(Goudie 2014; Haase 2003; Haase and Gläser 2009; Tockner et al. 2005; Ward et al. 2016)
	Hydrology	Distance to river ≤5 km	(Tockner et al. 2005; Ward et al. 2016)
	minus (Wetland HLU)		
Forest	Soil Type	Brown earth, pelosols, pseudogley	(Umwelt Bundesamt 2014)
	Slope	2:1 Ratio (50%)	(Urban et al. 2000)
	minus (Floodplain + Wetland HLU)		

### Linking Ecosystem Services and Hydromorphological Landscape Units

In order to find the ES values and patterns of the three HLU types, we combined the ES assessments with the resulting HLU delineation in a spatial hybridization approach. First, a spatial comparison was done of each of the mapped ES with the spatial extent of each individual HLU type. We draw on Haase and Gläser (2009) for the identification of ecosystem areas and combine established ES mapping methods in order to identify the state and spatial patterns of the identified areas in regard to their ES delivery. Each ES raster map was overlaid with each HLU map mask in order to obtain the mapped ES delivery of each area type. Second, the raster layer statistics were found for each of these maps in order to obtain the average ES value of each NBS location type. These results were visualized graphically. Multifunctionality is often seen as an important trait of NBS; thus, the ability to understand how these areas are behaving in terms of ES delivery can aid in developing and planning for trade-offs and synergies (Eggermont et al. 2015; Kabisch et al. 2016; Nesshöver et al. 2017; Turkelboom et al. 2018).

In order to have a better understanding and visualization of the varying ES delivery of each NBS type, all quantitative data for the ES delivery of the delineated HLU was then normalized to values from 1 to 10, i.e., new min and max, according to Han et al. (2012) using the following equation:

$$v^\prime = \left( {\frac{{v - min_A}}{{max_A - min_A}}} \right) \times \left( {new\_max_A - new\_min_A} \right) + new\_min_A$$

Normalizing the data looks to give results the same weight so that they fall within the same range. This looks to avoid dependence on the measuring unit and preserves the relationships among the original data values.

Additionally, rather than focusing on maximizing values or on exact values, we look to identify possible NBS areas where there is a low ES delivery present, as well as areas where a higher ES delivery occurs. In order to identify these areas, we derived the average value of each ecosystem service for each HLU type and used this as a basis of comparison to spatially identify areas of higher than average and lower than average ES delivery specific to each ES. That is to say, coinciding areas where three, two, or one ES were above average were spatially intersected and depicted, as well as areas where all three ES were below their corresponding average. It is important to note that multiple ES comparisons run the risk of unintended situations, where strengthening or focusing on one ES can result in the reduction of one or more of other ES (Aylward et al. 2005). While a win-win scenario is ideally sought, the reality is that trade-offs between options are ubiquitous and exact calculations and utility maximization do not necessarily solve the conflicts (Nesshöver et al. 2017; Turkelboom et al. 2018).

## Results

The results of the delineation and ES delivery of the NBS areas were spatially mapped and graphically presented. Additionally, the average ES values for the NBS areas were assessed and mapped to identify areas of trade-offs and synergies in order to further visualize the spatial patterns and critical locations.

**Fig. 3:**
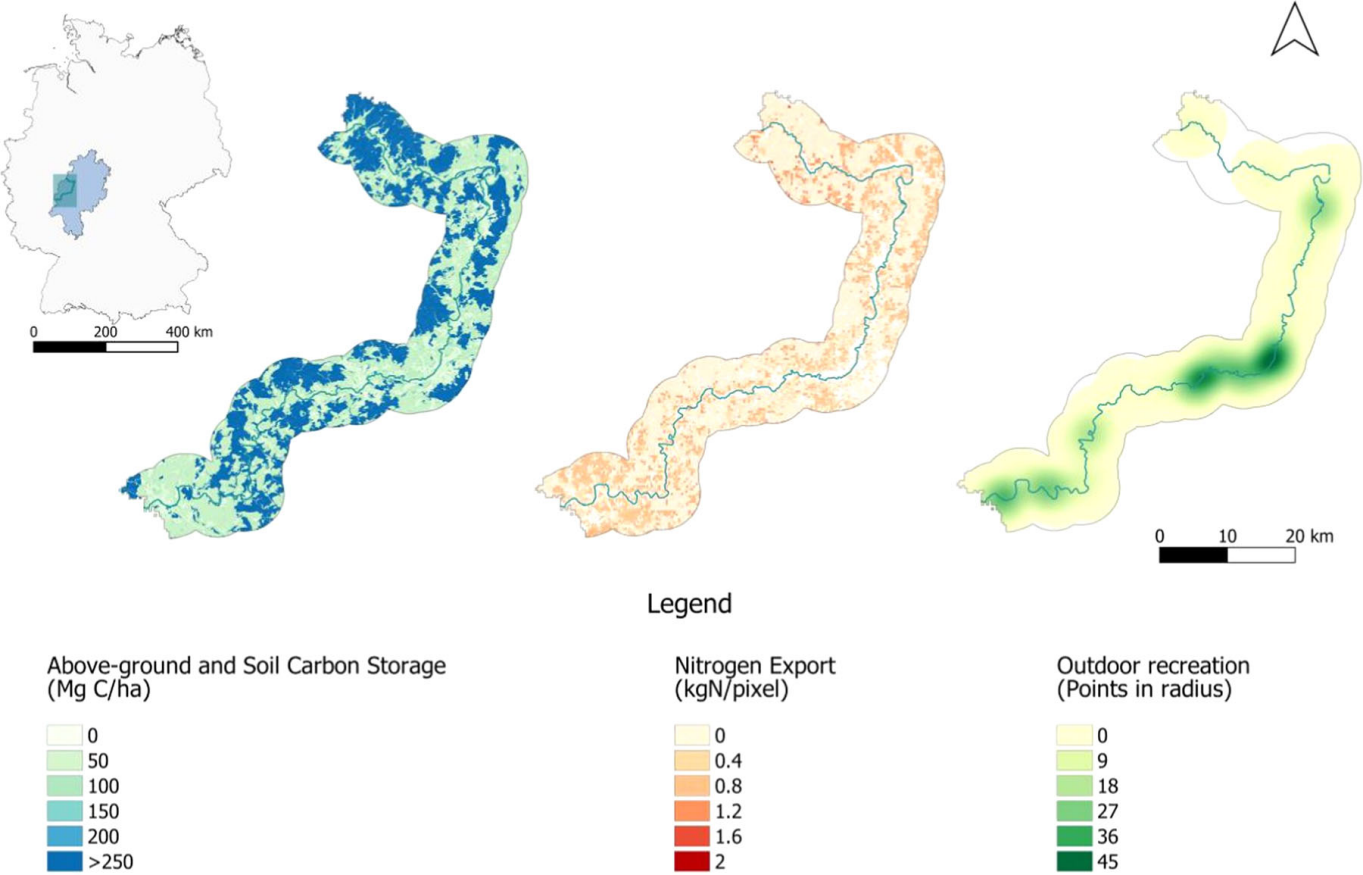
Ecosystem service delivery maps for the Lahn River stretch

### Ecosystem Service Indicators and HLU Delineation Maps

Figure 3 shows the results for the spatial pattern and values for each of the ES indicators used in this study. Both the above-ground and soil carbon storage and nitrogen retention indicators reveal a noticeable spatial heterogeneity present. In contrast, when examining the outdoor recreation indicator, core areas are identified. Here we note that the final output map of the NDR model shows the contribution of vegetation to purifying water by retaining nutrient pollutants from runoff. Thus, areas of higher values represent areas where nutrient export is higher and where pollutants are less apt to be retained.

**Fig. 4:**
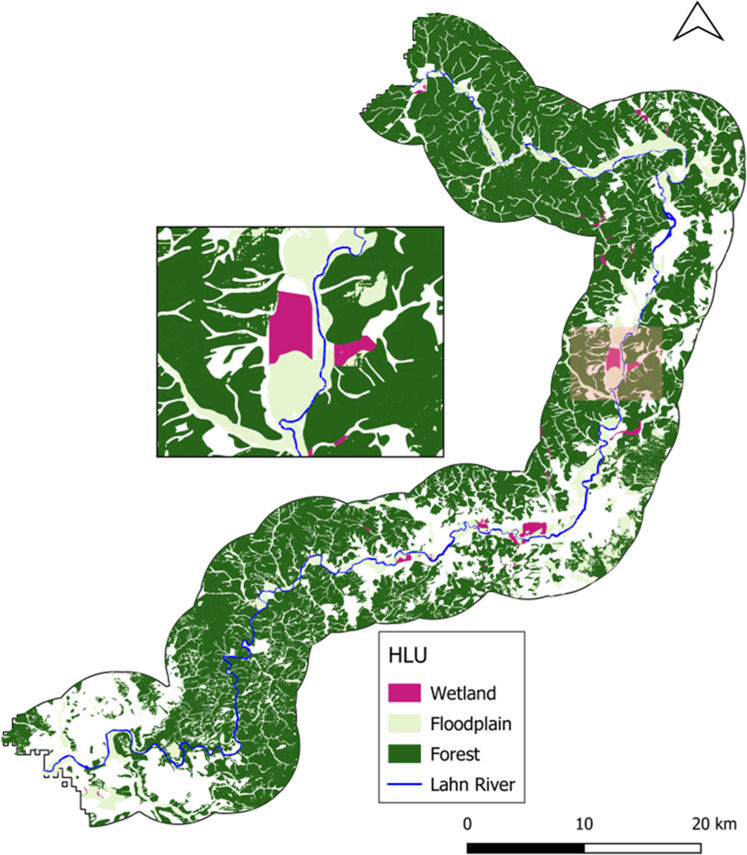
Mapped spatial extent of the hydromorphological land units, HLU, for wetlands, floodplains, and forests

The delineation of the three types of HLU resulted in maps of a landscape where all three natural features are present throughout the stretch of the river, see Fig. 4. As expected and explained by the very specific biophysical parameters, wetland areas are the natural features covering the least terrain, while floodplains and forest follow correspondingly. Specific statistics on the HLU results can be found in Appendix E.

Land uses currently under an urban or commercial agriculture use are not presented in this map as this map is the result of just the delineated natural features. These are land uses which reflect a human-driven use of the land (Haase and Gläser 2009; Schindler et al. 2016). In contrast, the HLU identification relied on natural parameters, such as soil type present and slope variation in the area. Admittedly, some of the areas identified as a wetland, floodplain or forest may be under a current urban or agricultural use. However, one can postulate that these kinds of areas contain the biophysical, natural characteristics which would allow for potential conversion to the corresponding NBS type.

### Spatial Patterns and Values of Ecosystem Services and Nature-Based Solution Types

**Fig. 5:**
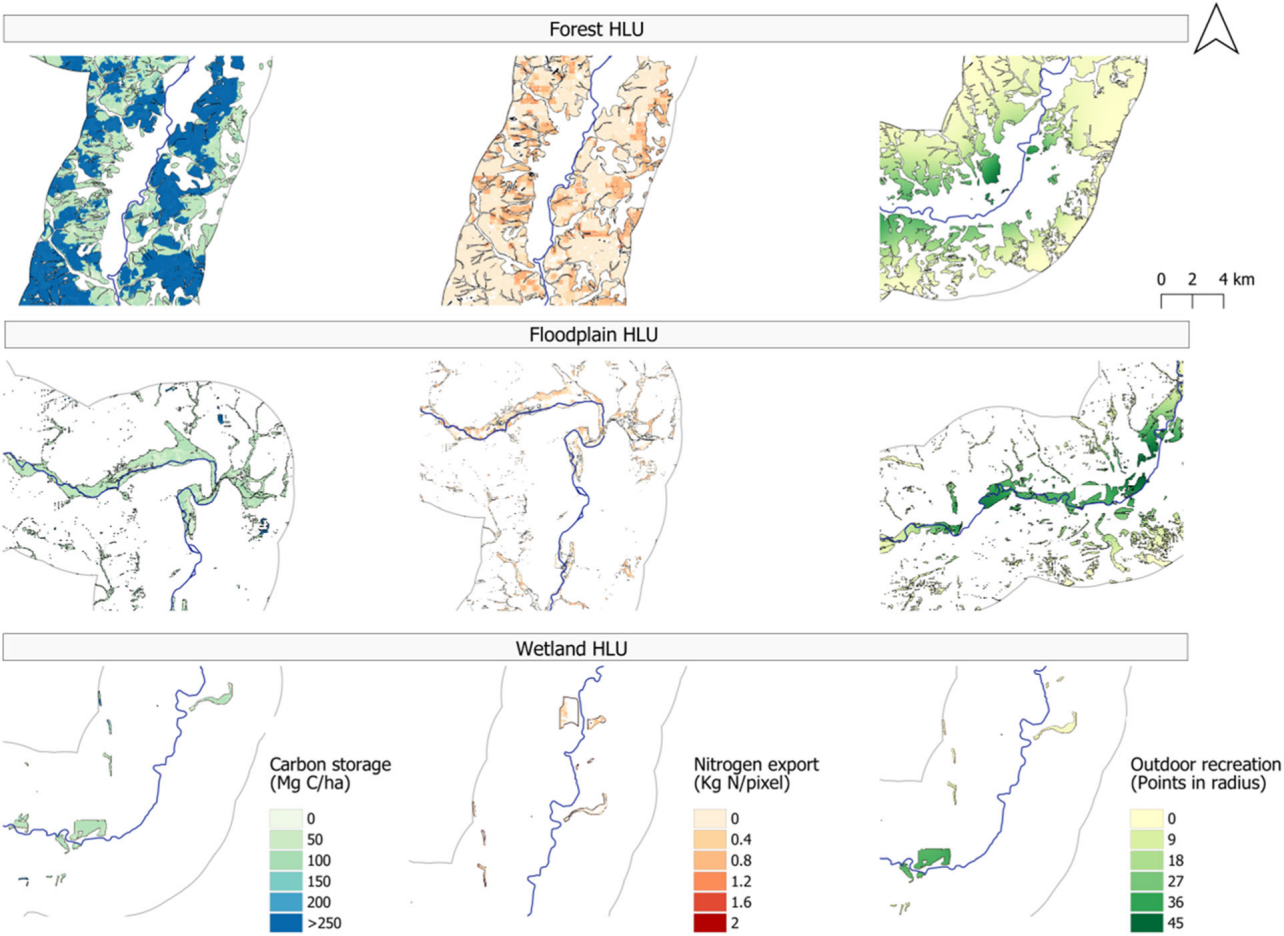
Ecosystem service values according to each HLU type

Figure 5 presents the distribution of the ES mapping results demarcated by the type of natural feature providing the service in question. The maps show the variability in ES values for each type of HLU. The presented focus areas are representative visual examples of the entire case study area for each HLU in question.

**Fig. 6:**
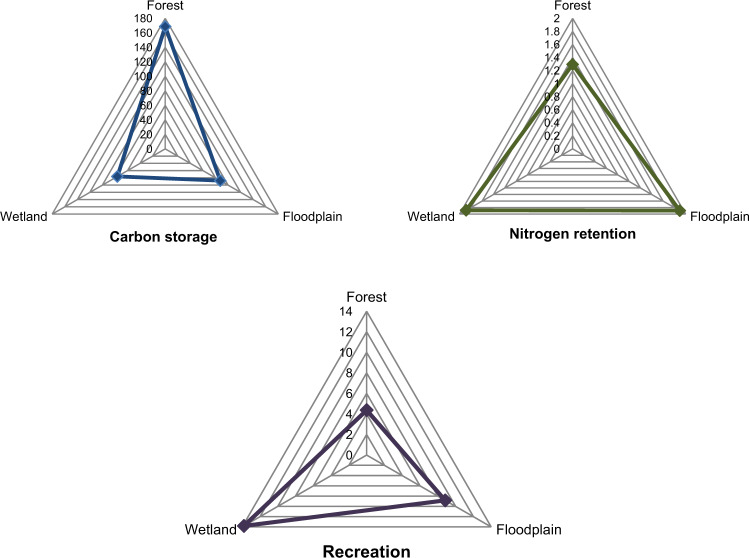
Average carbon storage, nutrient retention and recreation value per cell unit according to Nature-based solution type

In addition to the mapped results, the ES delivery values of the three NBS type areas were graphically analyzed. For the nitrogen data, the nitrogen load was compared to the nitrogen export in order to find the quantitative measure of the nitrogen retention service of the landscape, as per Sharp et al. (2016). Figure 6 displays the average ES delivery value per cell unit for each NBS type. A distinct composition of ES delivery can be observed for each NBS type.

**Fig. 7:**
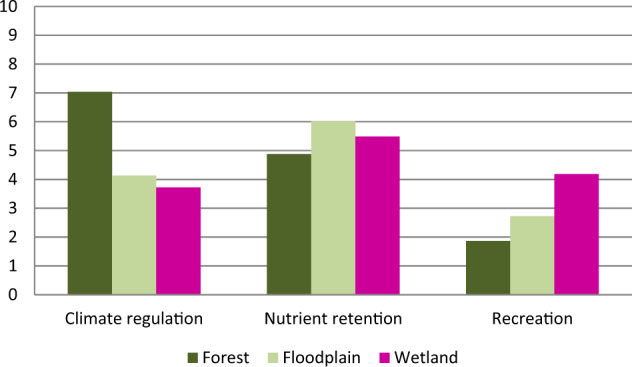
Normalized average ecosystem service delivery for the different possible NBS types (values are normalized between 1 and 10, where 0 presents the lowest and 10 the highest value)

The average for each of the ES quantified values corresponding to the possible NBS type was then normalized to allow for a facilitated comparison of ES delivery, see Fig. 7. In the study area and in regards to carbon storage, forest areas provide the highest level of carbon storage, followed by floodplains and then wetlands. The nutrient retention ES is similar for all three NBS types, yet floodplains tend to provide a higher delivery of this ES. Finally, recreation delivery is led by wetland areas, followed by floodplains and forests. These results should be carefully interpreted as they are based on the local, current state, and on average values. These results do not represent maximum values nor can they be read as a result for NBS types in general, i.e., other case studies.

The average ecosystem service value for carbon storage, nutrient retention, and recreation corresponding to each possible NBS type was used as the basis of comparison for the geospatial synergy and trade-off analysis, see Table 3 for specific values. This comparison drew upon Haase and Schwarz (2012) to distinguish between trade-offs and synergies. Rather than a temporal change in ES delivery, the focus in this research is sole of the status quo of the delivery of ES within a spatial realm. Thus, we focus on identifying the areas presently under a state where the ES delivery is above- or below average and coincides with the other ecosystem services. For this research, a synergy, i.e., a coincidence of high ES delivery, occurs when all ES are being provided at an above-average level, and a trade-off entails areas where one, two, or all three ES are being delivered at a below-average level.

**Table 3:** Average ecosystem service values according to HLU

HLU	Carbon Storage (Mg C/ha)	Nitrogen Retention (kg N/pixel)	Outdoor Recreation (Activity points/pixel)
Forest	168.78	1.29	4.38
Floodplain	87.67	1.90	8.82
Wetland	76.12	1.88	13.80

**Fig. 8:**
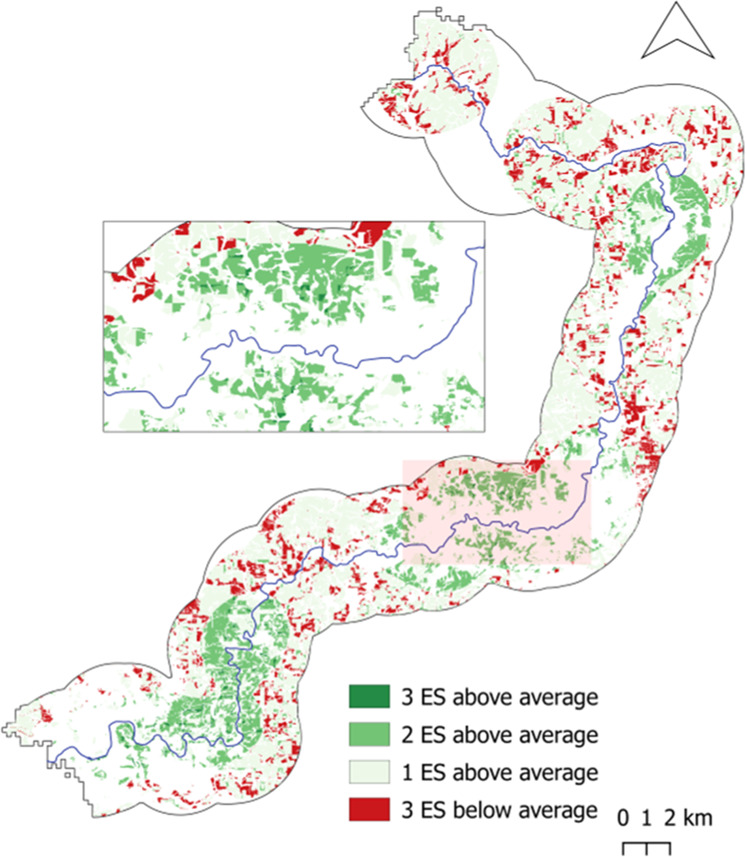
Map of coinciding above- and below-average ecosystem service delivery areas for Forest HLU

**Fig. 9:**
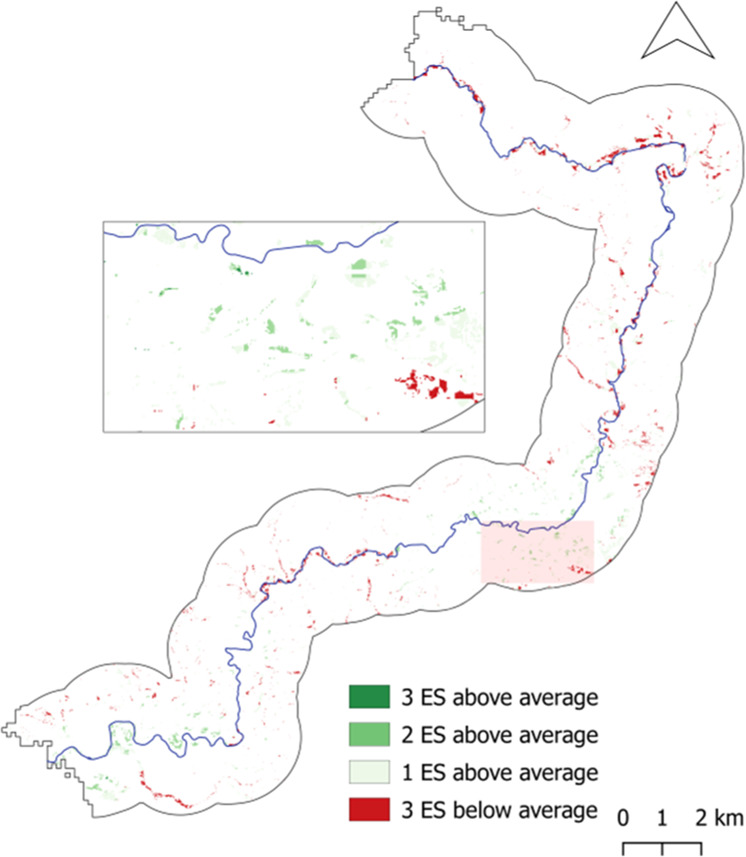
Map of coinciding above- and below-average ecosystem service delivery areas for Floodplain HLU

**Fig. 10:**
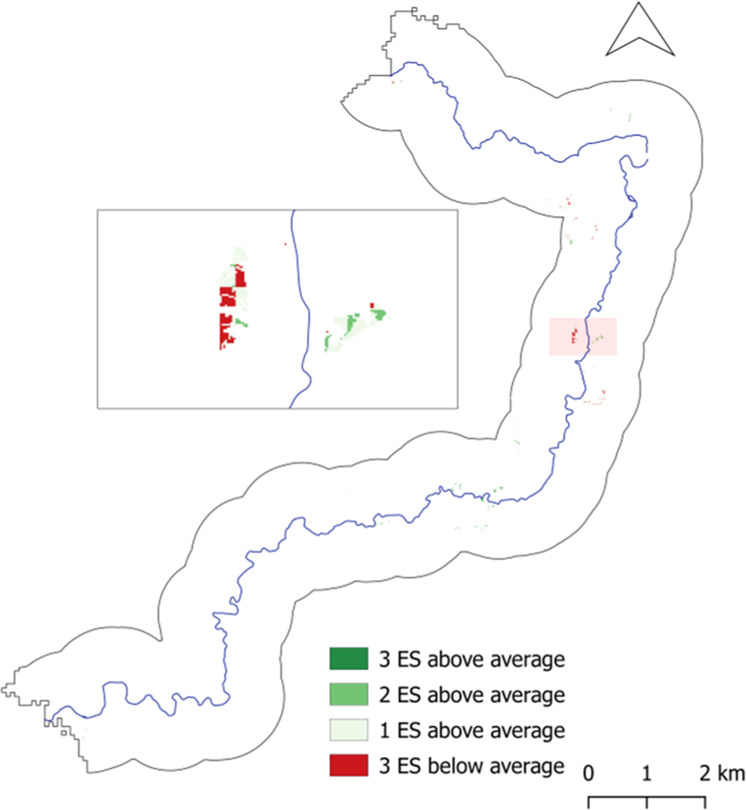
Map of coinciding above- and below-average ecosystem delivery areas for Wetlands HLU

Figures 8–10 present the results of this analysis according to each HLU type. These dark green areas are areas can be considered areas where there is a multifunctional river basin present in terms of the delivery of the three ES, i.e., areas where all ES are provided at an above-average level. Thus, these are areas of high ES delivery. Conversely, areas, where a low ES delivery coincidence was present, were identified. Here we focused on identifying areas where one or more of the ES values were below average. These are areas where there is an ecosystem service, or more, which is not able to reach a higher delivery level. These areas are likely in need of NBS measures and merit further analysis in order to identify what may be hindering the under-delivered ecosystem service(s). This notably applies to areas in dark red in Figs. 8 to 10, which present the areas in which all three ES are at a below-average delivery level. The legend entails for medium green that two ES are above-average, while one is consequently below-average. This also applies to the light green tone which illustrates one ES at above-average delivery and two ES below-average.

## Discussion

This research utilizes the delineation method to spatially identify areas of opportunity spaces for NBS development and of working NBS; thus, where NBS measures could be implemented, restored or should be safeguarded. It is an explorative approach which examines the use of ES as instrument to support NBS planning and implementation. Assessing the ES of possible NBS areas, strengthens the notion that ecosystem services can be a medium for which to facilitate understanding and communication of the effects and behavior of possible NBS (Albert et al. 2019; Nesshöver et al. 2017). Considering ecosystem services lie at the confluence between social and environmental systems and research, nature-based solutions are poised to benefit from this interface (Lavorel et al. 2017; Nesshöver et al. 2017). This research takes up this notion and presents results that allow for quantitative analysis and visual comparisons.

As illustrated by the results, the three ecosystem areas, wetlands, floodplains, and forests, are often complementary in terms of their ecosystem service delivery. With focus on the average ES value, each area delivers a distinct ES pattern and the highest delivery value is achieved, not by the same type, but rather each one has a distinct maximum. Therefore, supporting or enhancing a certain NBS measure will not necessarily decline the performance of the other areas when considering ecosystem service delivery. The use of HLU proves useful as an analysis entity which, does not just highlight visible spatial and ES patterns of the NBS spaces, but also allows for an understanding of the potential means to support ES delivery. This allows planners to contemplate a multi-functioning landscape with distinct NBS measures in place.

The delineation method identifies homogenous, biophysical units on a landscape scale with the intention that these units may help identify areas where multiple, rather than sole, NBS measures can be implemented. In other words, the results provide a landscape perspective of the spatial patterns of spaces with the potential to host NBS measures and the characteristic of their ES delivery. It does not focus on individual, isolated cases. For example, for the areas identified as wetlands, we recommend planners not just consider the individual unit as a possible site for measures, but rather that they acknowledge the broader wetlandscape, which encompasses natural processes and functions (Thorslund et al. 2017). Actual planning of NBS measures in practice should consider a landscape perspective and consider a range of principles including evidence-basis, integration, equity and transdisciplinarity (Albert et al. 2020, 2019). This delineation methodology builds upon the mapped historical floodplains in Haase and Gläser (2009), is inspired by water models and incorporates biophysical characteristics in the identification procedure. We looked to further a simple land-use denomination of a natural area with additionally defined criteria to have a more concrete understating of where potential NBS measures could be enacted. Other means to define the natural areas, or HLU, could have included different characteristics, such as perhaps precipitation data or social dimensions; yet, we chose the most apt, considering biophysical, natural focus, scientific correctness, and spatial analysis and dimension.

As mentioned, the delimitation exercise is based on the explicit biophysical criteria and does not include land use. The ecosystem service assessments focus on the status quo. Thus, each identified HLU area in terms of its actual land use is neither analyzed, nor its potential ecosystem service delivery. That is to say there is no scenario analysis to form a prognostic of what the ES delivery would be if all the areas were under a different land-use or used to their maximum “natural” capacity. These initial values are apt as a comparison medium for the planning of NBS, i.e., identifying spaces where NBS measures are possibly suitable and the current ES delivery state of that area.

The geospatial comparison analysis of areas of higher- and lower-than-average ES delivery offers an explorative approach to further understand where areas of focus for possible NBS measures implementation could occur. The results are auspicious, yet they should be very carefully interpreted. In addition to the limitations discussed in this section, such as data processing aspects, which create a fragmented picture of the areas, there should be a further assessment of what exactly is occurring at the locations, i.e., what the actual nature, and not the processed data, presents. For example, results for all three natural features show that coinciding areas of above-average delivery of all three ecosystem services are rarely present. Yet, identifying areas where all ES are above average values may not necessarily be the ideal situation. For example, it may be the case where it is best for wetland areas to have a low human presence, and thus, a low recreational service value while being able to provide a high nitrogen retention service. While the results do provide a more robust understanding of the distinct ES delivery pattern for the NBS spaces in question, the data should be carefully interpreted when speaking of trade-offs and synergies. The ES values presented here are meant to help to identify patterns, directions, and behaviors of NBS spaces for planning decisions and are not intended to interpreted as absolute values. By providing a variety of ES values, it may give the false impression that all ES “can be met at the same time,” yet, the reality is that “in most situations, it is impossible to manage ES in such a way that all these ES are simultaneously utilized at desired levels” (Turkelboom et al. 2018). Conflicting land uses and interest are part of the many variables in decision-making, and neither utility maximization nor strict calculus will solve these trade-offs (Nesshöver et al. 2017). It is recommended that additional methods and approaches, including socio-ecological, qualitative, hybrid, and experimental methods, be integrated in order to create a more robust knowledge for decision-making and planning (Lavorel et al. 2017; Nesshöver et al. 2017).

The method presented in this paper offers a strong natural science perspective for planners, yet remains simple enough in application. Unlike simple proxy or expert-based tables and models, this research combines carefully selected biophysical criteria based on established literature in order to define areas for possible NBS implementation. Considering that HLU entails multiple and solely biophysical components, this approach supports the spatial identification of natural areas and produces more defined and robust results in comparison to practices such as a land cover/land use, which are based solely on present vegetation. The fundamental biophysical focus of this research allows researchers to incorporate natural characteristics in assessments leading to a more accurate examination of what nature is doing (Lavorel et al. 2017). For a more coherent understanding of NBS, further incorporation of ecological systems and functions should continue to guide future research, as well as for ES models and assessments (Lavorel et al. 2017). The data, programs, and models used in this research are either open-sourced or upon request are made available to the public; this promotes an ease in transferability to other case studies.

This research incorporates relevant variables, such as soils and vegetation, in the HLU methodology in order to underscore key biophysical criteria in the ES assessment. Phenomenological models, such as the InVEST NDR model are also used; these models include semi-quantitative relationships, in order to move beyond look-up tables (Lavorel et al. 2017; Sharp et al. 2016). These types of models and mixed methods add robustness and reliability to the ES mapping. With this in mind, one must still consider the drawbacks of such methods and note that the ES values presented should be construed as the spatial variation and importance of the effects, rather than as absolute values.

Recognizing this research did not focus on furthering the ES assessment field, it does, however, call for utilizing ES assessments that go beyond simple land-cover information (Lavorel et al. 2017; Tomscha et al. 2017). While ES mapping and spatial distribution have advanced in the last decade, notable challenges such as scientific understanding, biophysical realism, data quality, remain a tremendous challenge (Brown and Kyttä 2014; Burkhard and Maes 2017; Lavorel et al. 2017; Seppelt et al. 2011). The InVEST NDR model considers biological mechanisms underlying ES supply and integrates land configuration, yet due to the number of assumptions, for these types of models, the direction or variation of a ES effect, rather than the precise value, should be considered (Lavorel et al. 2017; Sharp et al. 2016). The outdoor recreation data stem from PPGIS data which is often affected by the address of the participant influencing the mapped locations; furthermore, studies also show that higher geographic participation often reflects higher population densities (Brown and Fagerholm 2015; Haklay 2013). This explains the higher value areas closer to population hubs in this research. This coincides with Rall et al. (2017) and Radford and James (2013) which found that outdoor recreation decreases from an urban to rural gradient.

As this is an explorative research, there are evident uncertainties and limitations. In terms of uncertainty, the results themselves have not been validated in-situ, as the results are data and input dependent. Additionally, the verification of the HLU delineation was not in the scope of this manuscript, yet it is recommended for future research. The use of GIS data presents well-grounded, accessible, and visualized results; however, critical shortcomings such as pixelation and rasterization issues arise (Burkhard and Maes 2017; Neuendorf et al. 2018). At times, the conversion of vector layers to rasters entailed a loss in quality and precision and the created units may be visibly fragmented. This is also due in part to the heterogeneity of the landscape itself, which, due to many factors such as distinct land-uses and ownerships, creates situations, specifically for analysis in this case, where natural un-fragmented delineations are hard to attain.

Many of the insights emerging from the paper, including the benefits of establishing wetlands, resonate well with current discourses at local, regional, and state levels in the case study area. However, substantial barriers such as available space, funding, and the establishment of suitable governance models still need to be addressed to advance greater use of NBS opportunities in practice. As an explorative glimpse of the landscape which incorporates specific environmental parameters, this research can guide an initial decision-making for planners and interested parties, yet it should be accompanied by social, economic, and stakeholder analysis and considerations (Albert et al. 2020; Nesshöver et al. 2017).

## Conclusion

In the present paper, we present an explorative approach that advances the understanding of NBS through the use of HLU and consequent ES assessments. By applying mixed methods including established ES models and inspired by hydrological models, we have shown that the ecosystem services of areas apt for NBS can be assessed, compared, and present distinct spatial and quantitative patterns. The use of HLU as a basis for ES assessments of the opportunity spaces proves to be a useful medium for analysis. The results provide an initial foundation to foster discussion of NBS implementation in river landscapes between science, planning, and stakeholder groups. NBS has the potential to shift the river management paradigm and redirect the efficiency-focused, single-objective management toward one with more ecological and multifunctional strategies. Yet, further research in the ecosystem services field which closes the biophysical gap, as well as research focusing on understanding and quantitatively assessing the relation of NBS and their ES delivery is required in order to offer more scientifically sound results which planners, policy and interested parties can understand and utilize.

## Data Availability

As stated in the text when citing the corresponding sources.

## Appendix A. Input data and sources (BKG 2017; Fick and Hijimans 2017; Gottwald and Stedman 2020; HLNUG 2018; HLNUG Abteilung Naturschutz 2016)

**Table:**

Data	File Type	Source	Scale
Digital Elevation Model and Precipitation	Raster	WorldClim	30 m × 30 m
Soil Map	Shape	Office for Nature, Environment, and Geology of the state of Hesse (HLNUG)	1:50,000
Watercourse and Watershed	Shape	Federal Agency for Cartography and Geodesy (BKG)	1:50,000
Land Cover Germany (2013)	Shape	Federal Agency for Cartography and Geodesy (BKG)	1:50,000
Vegetation	Point	Natis data	1:50,000
Groundwater Levels	Point	HLUG Conservation Department	NA
Recreation	Point	Lahn valley Maptionnaire Open Survey Results	NA

## Appendix B. Indicator values for each land use class adapted from (HLUG 2005; Majidzadeh et al. 2017; Strohbach and Haase 2012; Umweltbundesamt 2018) for carbon and (Knoll et al., pers. comm. 2019) for nitrogen loads

**Table:**

River ES		Carbon Storage		Nutrient retention	
Land-use nomenclature	Above-ground carbon storage (MgC/ha)	Soil carbon storage (MgC/ha)	Total carbon storage (MgC/ha)	Total nitrogen load (kgN/ha)	Nitrogen efficiency
Continuous urban fabric	4.20	21.32	25.52	10	0.05
Industry and retail	8.52	22.33	30.85	10	0.05
Mining and vegetationless sites	0.74	28.42	29.16	10	0.2
Mixed urban fabric	20.00	28.42	48.42	10	0.2
Transportation	2.6	22.08	24.68	10	0
Cemetery	27.84	82.30	110.14	10	0.5
Park and leisure activity area	24.38	102.00	126.38	10	0.4
Agriculture	4.81	59.77	64.58	55.9	0.6
Grassland	3.78	77.43	81.21	5	0.5
Complex cultivation	16.03	111.70	127.73	5	0.6
Hardwood forest	68.31	175.80	244.11	5	0.8
Coniferous forest	72.91	175.80	248.71	5	0.8
Mixed forest	75.71	175.80	251.51	5	0.8
Transitional forests	10.12	73.18	83.30	5	0.8
Wetland and Reeds	98.26	74.00	172.26	5	0
Water	0.00	0.00	0.00	0	0

## Appendix C. Values used for InVEST NDR according to recommendations from Redhead et al. (2016)

**Table:**

Variables	Selected
Critical length	25
Subsurface N proportion	0
TfA	100
Borselli k	2
Subsurface critical length	0

## Appendix D. Outdoor recreation activities types and examples of activities used for this paper, as in the previously conducted PPGIS survey in the Lahn river by Gottwald and Stedman (2020)

**Table:**

Outdoor recreation activities	Examples of activity types
Nature activities	Hiking, fishing, observing nature, experiencing landscape or other.
Recreational activities	Walks on the shore, walking the dog, passenger ship, rest and relax, cultural activities, other.
Sport activities	Cycling, running, walking, canoeing, kayaking, rowing, sailing, surf (wind, kite, stand-up paddling), other.

## Appendix E. Hydromorphological landscape unit area data

**Table:**

HLU Type	Total Number of Units	Area (ha)				
		Total	Maximum	Minimum	Average	Median
Wetland	62	907.05	189.81	<1.00	14.63	2.91
Floodplain	10463	10776.45	594.62	<1.00	1.03	0.06
Forest	3956	77174.37	2121.38	<1.00	19.51	5.75
